# Cross country lessons sharing on practices, challenges and innovation in primary health care revitalization and universal health coverage implementation among 18 countries in the WHO African Region

**DOI:** 10.11604/pamj.2022.41.159.28913

**Published:** 2022-02-23

**Authors:** Humphrey Karamagi, Regina Titi-Ofei, Michelle Amri, Sosthene Zombre, Hillary Kipruto, Aminata Binetou-Wahebine Seydi, Gertrude Avortri, Juliet Nabyonga, Prosper Tumusiime

**Affiliations:** 1 WHO Regional Office for Africa,; 2 Dalla Lana School of Public Health, University of Toronto, 155 College St, Toronto, Ontario, M5T 1P8, Canada,; 3 Takemi Program in International Health, Harvard School of Public Health, Harvard University, 665 Huntington Avenue, Bldg 1, Room 1210, Boston, MA, 02115-6021, USA

**Keywords:** Health policy, health planning, health systems, development

## Abstract

The fifth health sector directors´ policy and planning meeting for the World Health Organization (WHO) regional office for Africa convened to focus on building health system resilience during the COVID-19 pandemic to ensure continuity of essential health services, primary health care (PHC) revitalization, and health system strengthening towards achieving universal health coverage (UHC). In this paper, we present short summaries and experiences shared by 18 countries, for which their practices and outcomes have been documented in this manuscript. These actions are aligned with six key themes: (i) defining and making more essential health services available, (ii) increasing service coverage targeting hard to reach populations, (iii) financial risk protection, (iv) improving user satisfaction with services, (v) improving health security, and (vi) improving coverage with health-related sector services. It is through these shared country experiences that lessons are learned that can influence the region´s work and advancement to achieve UHC through a PHC approach.

## Introduction

Universal health coverage (UHC) is central to the 2030 sustainable development agenda, and the employment of a comprehensive PHC approach is critical for its attainment. Looking to the heightened prominence of UHC, entailing “all people have access to the health services they need, when and where they need them, without financial hardship” [1], COVID-19 has allowed us to ask: “Will future policy reflect lessons learned, to not only mitigate risks from further crises, but also tackle many other policy challenges?” [2]. The recent WHO AFRO regional forum on health system strengthening for UHC and SDGs sought to shed light on this question, providing a platform for deliberation on the emerging issues relating to health systems development in the context of UHC and other health related SDG targets. It provided an opportunity for cross-country peer learning, experience sharing, and assessment of the direction of systems development amongst countries of the region [3]. At this meeting, directors of policy and planning shared actions taken to strengthen the PHC approach in moving towards UHC. These actions and subsequent outcomes are detailed by country in this manuscript.

The influence of piecemeal initiatives remains limited to catapulting action towards attainment of UHC and other health related SDG targets [4]. Such platforms as this regional forum provide an opportunity to break down some of the traditional siloes and barriers, that may often present bottlenecks to facilitate such exchange processes, as countries work together towards the 2030 agenda. Through the fifth health sector directors´ policy and planning meeting, the WHO African Regional Office facilitated such exchanges, aligning them with a set of health system outcomes as defined in the region´s framework for health systems development towards UHC [5,6]: (i) defining and making more essential health services available, (ii) increasing service coverage targeting hard to reach populations, (iii) financial risk protection, (iv) improving user satisfaction with services, (v) improving health security, and (vi) improving coverage with health-related sector services]. Countries were divided across these themes, to then share actions and practices undertaken within the country and associated outcomes. This manuscript aims to elevate this knowledge exchange one step further, through translating country actions into a case study, to afford exchange across countries with varying contexts, and socio-demographic influences. “It is through establishing common ground, strategizing together, and taking small steps, that we are engaging in “re-organized action” - coming together to reorganize by thinking through our preconceptions [...] and clarifying next steps for action [....]” [7]. This paper highlights the practices and experiences shared by countries. It highlights countries where practices and their observed outcomes were shared, and also highlights countries where specific challenges have presented bottlenecks for progress towards improved system outcomes, as well as the steps that are being taken to address these. While this exposition is largely descriptive, and does not provide a judgement of country performance against these outcomes, it provides a non-biased overview of the issues raised by countries on the innovations, actions, and policies that have been employed on the road to UHC attainment.

## Workshop report

**Case study: country lessons shared**

**Defining, and making more essential health services available**

**Burundi**

*Practice*: Burundi held a national conference on primary health care revitalization on 16-18 September 2019 to take stock of the implementation of the country´s commitments related to PHC and the health-related SDGs.

*Outcomes*: the government, in collaboration with its partners, made relevant recommendations with the establishment of a unit to monitor the implementation of these recommendations at the presidential level. These include: focusing on leadership and governance to improve intra, inter, and multi-sectoral coordination, international aid, develop a health in all policies strategy, and others; ensuring sustainable, quality, integrated and person-centred PHC that takes into account the needs of individuals, families, and communities in all policies is relevant; improving health human resources through strengthening training, ensuring equitable distribution of providers across the country, and ensuring the strengthening of mechanisms for the remuneration, motivation, and retention of specialized personnel; and ensuring individual and community empowerment through their participation at all stages of public health interventions, joining the UHC global compact 2030, establishing multi-stakeholder platforms to ensure heightened participation in regular policy dialogue, strengthen the performance of community health worker groups and other community-based associations, and others.

**Cameroon**

*Practice*: established the UHC national technical group in 2015 and performance-based financing (PBF) was implemented to improve the coverage and quality of maternal and child health services by purchasing quantity and quality bonuses from health facilities. This has been ongoing since 2012 with 14 pilot demonstration sites in three regions (North West, South West and East) and implemented in the 10 regions of the country. The focus has largely been on maternal and child health and adolescent health indicators (HIV/AIDS, etc.), among other key country indicators. Interventions implemented included: signature of performance contracts; purchase of cover; quality control by higher level stakeholders; and common verification.

*Outcomes:* performance-based financing (as determined through the evaluation conducted) found that it improved: maternal and child health vaccination rates (which are now over 17%), direct payment of radiology and laboratory fees (-1473.44 Central African Francs), direct payment of illegal service fees (-2254 Central African Francs), quality of services with respect to the technical platform, and the overall satisfaction of women (improving 8.6% points). However, no improvements were recorded for the quality of care in terms of technical and interpersonal quality.

**Comoros**

**Practice**: Comoros established a vision (2015-2024): “an efficient national health system that enables the entire population, particularly the most vulnerable and disadvantaged, to have access to quality health care, with the effective involvement of all public and private actors and stakeholders, in a spirit of solidarity, equality, equity and social justice”. In alignment with this vision, various initiatives and steps have been carried out, including: assessing financial flows; joining the International Health Partnership; receiving the commitment of the head of state; adopting a law establishing health insurance; hosting a capacity-building workshop; undertaking a feasibility study; revising the national pharmaceutical policy; undertaking construction for a 600-bed university hospital centre; providing free services to those with HIV, TB, leprosy and malaria; providing services targeting maternal and neonatal health, children under five years of age, those with neglected tropical diseases, and those with non-communicable diseases; and many others.

*Current steps for improvement and/or ongoing challenges faced*: despite the several actions taken, challenges remain, such as around: strengthening effective coordination and sectoral dialogue mechanisms; weak technical and institutional capacity of the national health information system; implementation of the legal and regulatory provisions of the new health code; development of the public-private partnership; weak mobilization of financing for the implementation of generalized health insurance; and implementation of a national community health strategy in alignment with the Astana declaration.

**Increasing service coverage targeting hard to reach populations**

**Algeria**

*Practice*: undertook a twinning study (instituted and regulated by the government) which sought to: provide care to patients in the southern regions and the highlands whose specialties and/or skills are in short supply; provide training to medical, paramedical, administrative and management staff on a cyclical basis; establish links and networks between institutions to organize the transfer of complex cases for their certified education program (CEP) at the pilot institution level; and avoid the displacement (travel) of patients to university cities, by having the teams move to these populations in these health structures which generally have the necessary equipment.

*Outcomes*: the work resulted in numerous twinning agreements and developments, including: 109 public hospitals, 101 agreements signed, and 45 agreements in the process of being signed. As well, embraced a strong stakeholder communication strategy (involving the media, internal meetings, and mobilizing the public) and drew commitment from the state with 100% matched funding. However, challenges remain around telemedicine and exchange programs which are increasingly developed in healthcare institutions to support remote local teams in helping patients with diagnosis or CEP and distance education for health professionals

**South Sudan**

*Practice*: South Sudan has implemented a community health strengthening strategy, the “boma health strategy”, which trained community health workers in over 55 counties deliver a package of health promotion, disease prevention, and selected curative maternal and child health services.

*Outcomes*: numerous successes have been realized, such as increasing access and demand for health services in the community. Due to the availability of health services within the community, families no longer have to walk long distances to access health services at distant health facilities, nor are they financially impacted, as these services are provided free of charge. Additionally, available health services (including drugs) have improved demand for services, more community members have utilized health services, and drug consumption has increased. Service delivery has been regular with minimal disruption due to continued partner support. However, challenges remain around: the sustainability of free services (unless there is political will to increase the proportion of the government budget allocated to health, which currently is 2%); limited ownership of the program by the community and local authority; quality of care (including not all facilities trained on infection prevention and control); need to adopt the patient safety strategy and associated development of guidelines and protocols; and select locations not yet fully implementing the service package.

**Republic of Congo**

*Practice*: establishment of the national health policy designed to improve equitable access of the population to packages of essential and quality services. For which the priority is to strengthen PHC, through the revitalization of the health districts to move towards UHC and achieve Sustainable Development Goal (SDG) 3 (health).

*Current steps for improvement and/or ongoing challenges faced*: an evaluation of WHO support to the ministry of health in the 12 districts in 2020 is underway and will show the evolution of the 18 indicators selected in the monitoring and evaluation plan. However, to-date, 50% of cases of yaws have been detected and notified by the community relays. The indigenous population is concerned about prevention and protection against all forms of violence. To address this, since 2017, 200 indigenous community relays have been trained in order to improve the social demand for the use of health services. Further, health workers in health facilities are aware of the principle and importance of non-discrimination against indigenous people in public and private health care structures. While equitable access to quality essential care and services for vulnerable populations (rural, peri-urban, indigenous) remains a challenge in the Republic of Congo, the country has embarked on a process of decentralization for the implementation of PHC to address this challenge.

**Financial risk protection**

**Ghana**

*Practice:* Ghana has worked towards achieving FRP through scaling up various interventions, including: electronic mobile phone renewal of national health information system membership, increased recruitment of community health nurses, improved claims reimbursement time, investment into health facilities at the primary level (hospitals and polyclinics) and nurses and midwives, supply chain management systems, and reforms (e.g. framework contracting to take advantage of economies of scale, last mile distribution, logistics management information system).

*Outcomes:* numerous successes have been realized in Ghana, such as: increased active membership in the insurance scheme (over 40.5% of the population), over 60% of beneficiaries in the scheme exempt from fees, improved health service utilization, over 90% of out-patient department services provided at the primary level (i.e. district hospitals, health centres, and community-level services), reduction in malaria fatality rate (from 0.51% in 2015 to 0.1% in 2019), and others. Additional successes have been realized in four key areas: physical access to essential health services, financial access to essential health services, demand for essential health services, and resilience of health systems.

**Rwanda**

*Practice:* Rwanda´s efforts have focused on financial risk protection (FRP) and have accordingly introduced a Community-Based Health Insurance (CBHI) scheme to improve access to healthcare services and reduce significantly out-of-pocket expenditures, in particular, for the poor and most vulnerable. 90% of the population was covered by health insurance (with CBHI coverage at 79% as of end of fiscal year 2018/19). Interventions that have been scaled up to facilitate improved FRP: insurance coverage for the informal sector through CBHI; moving from flat to stratified premiums according to socio-economic categories; fully subsidizing the poor and most vulnerable population in Ubudehe Category I; access to broadened benefit package over time (from primary care and limited district hospitals package in early development to specialized and tertiary care); reduced fragmentation of CBHI pooling risks from 31 pools to one single pool; and increased CBHI revenues for financial sustainability.

*Outcomes:* efforts on FRP have resulted in successes in four key domains: physical access to essential health services through improved health worker density, effective referral system, task-shifting of select services at the community-level, establishing non-communicable disease clinics in hospitals and health centres, and ensuring one health post in every administrative cell to reduce travel time; financial access to essential health services though increasing the sources of revenue for CBHI to ensure sustainability, full subsidization of select populations, moving from flat to stratified premiums by socioeconomic status, and others; demand for essential health services has increased, as the utilization rate for CBHI beneficiaries has increased from 0.3 to 2.05 visits per capita (from 1997-99 to 2018/19); and quality of care through pursuing accreditation programs, digitalization of health services through scaling up electronic medical records, improving claim management, and enhancing the pharmaceutical supply chain.

**Mauritania**

*Practice:* despite efforts to reduce the burden of current health expenditure on households in Mauritania, it remains at 52% of health expenditures as of 2017. Mauritania carried out specific interventions to address this. In 2020, a national program aimed at strengthening social security coverage and direct income for the most vulnerable families, financing: free resuscitation for all; a 55% reduction in the amount of the Obstetrical Package with the extension of the package to all the needs of pregnancy and postpartum and everywhere in public structures; free medical transportation during referrals and treatment of road accidents; and ongoing medical insurance for the poorest households on the social register.

*Current steps for improvement and/or ongoing challenges faced:* a program to improve social supply and support demand was launched by the public authorities in 2020, and is investing more than $45 million over 30 months through nine health projects to help reduce financial and geographic barriers to access to health services for the poorest, accelerate progress towards UHC, regulate the sector, strengthen geographic coverage, and improve health system resilience. This financing should be sustainable after 30 months by being integrated into the public financing of the sector in order to make progress towards UHC. An additional budget line of 7.7 million dollars has been included in the 2020 finance law to reimburse health facilities for expenses incurred for this free service. The anticipated outcomes include: reducing the share of direct payments in current health spending; contributing to the reduction of the poverty rate; and faster progress toward UHC.

**Sierra Leone**

*Practice:* while about 60% of Sierra Leoneans live below the poverty line, over 60% (2018) of total health expenditure come from households (while the government contributes around 4% and donors account for about 36%). As such, Sierra Leone selected to address the high out-of-pocket payments at points of service delivery. Accordingly, the Social Health Insurance Law was passed in 2017 and was to be implemented as a prepayment health insurance scheme with the government providing the major source of funding.

*Current steps for improvement and/or ongoing challenges faced:* various initiatives have been undertaken and are ongoing in Sierra Leone, including: completing the UHC Roadmap to be implemented through the National Health Strategic Plan, to make quality health care accessible to all, everywhere, and at all times; abolishing user fees for pregnant women, lactating mothers, children under five years of age, and Ebola victims under the Free Health Care Initiative; revisiting school health; engaging over 30,000 community health workers, especially in rural and challenged communities; increasing health human resources by 4,000 people (40%) and receiving approval for the recruitment of another 3,000; implementing mandatory social health insurance; engaging the community for partnership and ownership through the revised primary health care handbook; and continued commitment to the global quality of care network.

**Improving user satisfaction with services**

**Burkina Faso**

**Practice:** Burkina Faso undertook an evaluation to assess response to needs with attention to select characteristics: dignity; autonomy; confidentiality; prompt decisions; access to social support services; quality of basic amenities; and the choice of health care providers. Based on the analysis carried out, Burkina Faso adopted a reform of its healthcare system based on quality management, the integrated person-centered care approach (ASICP), and patient safety. Specific interventions carried out included: National Strategy Combining Quality, Country Policy and Institutional Assessment (CPIAA), and patient safety; ASICP tools; Regulatory Quality Charter; quality reference; certification/accreditation; community watch; management of adverse care events; traditional medicine interface; and community health.

*Outcomes:* the implementation of these approaches has improved access and demand through free health care, universal health insurance, taking into account the needs of users, the involvement of other profiles in health care (in particular health psychologists and sociologists), and the involvement of the population in self-management (especially of chronic diseases and in health education). Health care channels and networks have been created and strengthened. It also made it possible to strengthen the resilience of the health system for continuity of care in insecure areas and in the context of COVID-19. However, Burkina Faso experiences difficulties with the inclusion of people-centered care indicators in the reporting of the national information system.

**Eswatini**

*Practice:* following various client satisfaction surveys, a client satisfaction and feedback mechanism (CSFM) was developed and deployed in 2019 in all health facilities across the country to strengthen capacity of health managers to manage client feedback. It consists of questions in multiple choice form and responses are used by healthcare workers to improve the quality of the healthcare service provision. Between 4^th^ February and 31^st^ August 2020, a total number of 12,677 respondents/customers have given feedback. Outcomes: the CSFM has resulted in numerous outputs, including: customers satisfied with service provision (ranging from 85 to 90% between February to August), availability of drugs (ranging from 64 to 76% in this same period), average waiting time (with most responses indicating wait times of under thirty minutes, but ranging to more than two hours), cleanliness in the health facility (majority of respondents indicating very good and good), and attitude of health care workers (majority of respondents indicating very good). However, some challenges remain, such as some clients who are reluctant to share mobile numbers, no offline version in case of power/network interruptions, need to fund the CSFM through government, insufficient feedback mechanism to the client that submitted the issue of concern, and inadequate practice of “client first” culture in entire continuum of health care.

**Gabon**

*Practice*: Gabon undertook an assessment of user satisfaction levels to identify areas of dissatisfaction, in order to improve health system performance. The evaluation of the service's response to needs involves the following characteristics: dignity, autonomy, confidentiality, prompt decisions, access to social support services, quality of basic amenities, and the choice of health care providers. Gabon elected to focus on the reception of patients in health facilities, as users raised concerns about the quality of basic amenities, confidentiality, and wait times.

*Outcomes*: the Ministry of Health has therefore initiated several reflections to improve the quality and supply of care. As such, numerous interventions were introduced, including: adopting a law that considers the rights of patients, dissemination of this charter to all health facilities, creating centralized services to receive patients, establishing mobile screening units in all health regions, mass awareness campaigns, and others. This required investments made to improving essential health services through access, demand, resilience, and quality of care. Improvements measured included: mass awareness campaigns for several years during the month of October for female cancers has enabled the population to adopt more responsible habits for monitoring their health (practice of sports, healthy food, etc.); the realization of CAP and socio-anthropological studies on cancer and other pathologies has led to a better understanding of the determinants of the population's demand for care; mobile screening units considerably improved the geographical accessibility of patient care (up to the departmental level and in the villages); and others.

**Zimbabwe**

*Practice:* Zimbabwe implemented the first generation of RBF which was aligned with and supportive of the National Health Strategy of 2010-13 by design. This included: user fee removal to improve access and utilization of health services; rebuilding basic standards of health services delivery-capital investment in facilities; strengthening the referral system from primary to secondary; and a strong thrust towards PHC. The RBF was piloted in two front runner districts (Zvishavane and Marondera). After evaluation, it was scaled-up initially to 18 districts in 2012 and all additional 42 districts.

*Current steps for improvement and/or ongoing challenges faced:* the RBF has resulted in: enhanced capacity to accurately report and utilize data, decentralized planning and investments of subsidy earnings for performance improvement at facility and district levels, significant improvements in coverage of maternal and child health indicators (observed throughout the country in general and faster rates observed in rural RBF districts for key indicators), significant improvement in the quality of select antenatal care services, and notable improvements in uptake of vouchers in past quarters. However, not all indicators show relative improvement under RBF. In the future, additional attention should be afforded to: careful selection of indicators to maximize efficiency in spending, revisiting how quality of care is measured and incentivized under RBF, combine RBF with complementary investments in quality improvement, and involve community health workers through a package of indicators and enhance demand through various initiatives.

**Improving health security**

**Lesotho**

*Practice:* the main health security issue/policy problem Lesotho faced amidst COVID-19 was rising co-morbidities and excess mortalities from communicable and non-communicable diseases. This included the: inadequate integration of health system strengthening and health security efforts; inadequate coordination of efforts between ministry of health and partners; low level of community awareness and engagement; and weak information systems.

*Outcomes:* to combat this, Lesotho scaled up interventions to facilitate the protection of populations from health risks (e.g. integration between health system strengthening and health security investments; prioritization of individuals and populations with vulnerability; repositioning of skilled health workers, including mobilizing and motivating village health workers; monitoring and sustaining sufficient stocks of essential drugs (child, TB, ART, etc.) and vaccines). This work was due to successes with multi-stakeholder collaboration (as the partnership allowed for the identification of interventions and associated resource mobilization), which led to enhancing health security and better targeting susceptible populations.

**Guinea**

*Practice:* Guinea drew on experiences from the Ebola epidemic to make necessary investments to strengthen their health system. Some of these investments include: developing strategic frameworks, plans, and policies; setting up a coordination framework; strengthening community health; significantly increasing the number of health structures; increasing health human resources at the health and community structure level; increasing the health care budget; modernizing the National Health Information System; and strengthening the supply chain and system for health products.

*Current steps for improvement and/or ongoing challenges faced:* strengthening the health system has increased access, demand creation, resilience, and quality of care. However, challenges remain around maintaining the quality of health care and services and early recovery through state ownership.

**Democratic Republic of the Congo (DRC)**

*Practice:* established a strategic framework and undertook a review of essential health services, including: vaccination, maternal and child health, HIV/AIDS, bed net usage, and tuberculosis.

*Outcomes:* determined various successes, including: vaccination coverage is greater than 80% (as of early May 2020), more pregnant women received prenatal care from a trained health professional (88% in 2013 compared to 80% in 2007), childbirth assisted by a health personnel (80% in 2013 compared to 64% in 2007), households had and used impregnated mosquito nets (9% of households in 2007 compared to 70% in 2013), and decreased deaths due to malaria (despite more cases being recorded, pointing to increased reporting). Challenges remain around: health financial resources are limited and efficient use needs to be improved; frequent and health emergencies related to Ebola virus and the COVID-19 pandemic tend to inhibit progress and the ongoing work and interventions being undertaken to reach UHC; the persistent insecurity in various regions; and health in all policies, as health interventions and progress are dependent on other sectors.

**Senegal**

*Practice:* Senegal incorporated the Primary Health Care Performance Initiative approach (which integrated three domains of the health system: capacity, performance, and equity), adjusted their targets based on these various ODD as well as their strategic plan, and established a multi-sectorial compliance plan in the response to the COVID-19 pandemic to ensure continuity of health services (including establishment of a 2020-2024 sectorial investment plan).

*Outcomes:* the ministry of health and advocacy began improving the quality of healthcare services through various experiences at hospitals and health centers (*centres de santé* and the laboratories). This initiative is designed to assist and help develop an environment in which health system´s actors will know what to do in order to provide quality health care, have the competence to do so and that they stay engage with available resources. These numerous initiatives, policies and actions resulted in better health indicator outcomes, including a decrease in maternal mortality (236 for 100,000 live births) and infant mortality (37 for 1000 per live births, as of 2017). The ministry of health and advocacy will continue to improve PHC performance through developing more community approaches, multidisciplinary strategies, and using health information.

## Discussion

Cross-country experience sharing opportunities provide an opportunity for peer learning, experience sharing and assessment of the direction of systems development amongst countries of the region. It aims to build a common understanding, and knowledge on taking forward the UHC agenda in the region. Across the three six themes highlighted, it has emerged clearly that countries in the region are making progress towards attainment of these goals of improved health security; defining, and making more essential health services available; increasing service coverage targeting hard to reach populations; improving financial risk protection; and improving user satisfaction with services. Against the background of a rapidly changing context of health, driven by the COVID-19 pandemic, countries have all faced significant and un-anticipated challenge to their health system capacities. Together with this, the Astana Declaration on Primary Health Care and the adoption of the SDG 3 Global Action Plan all call for a re-think of the design, focus and monitoring of health systems development in the WHO Region for Africa. The experiences shared have demonstrated that countries are actively rethinking policies and actions to re-align with the current needs of their health sectors to make them fit for purpose in the current and future health environment and in regard of the overall 2030 agenda. Beyond these experiences sharing sessions, WHO AFRO continues to provide technical support to countries in health systems strengthening, through the UHC flagship program, and implementation of the regional framework for primary health care operationalization. One key aspect of this support, particularly in the context of the COIVID-19 pandemic, is the support for strengthening health systems to ensure continuity of essential health services. Tools including health facility assessments for readiness and service capacity have been implemented across the region, as well as support for embedding measures for assessing health service resilience in routine health information systems.

## Conclusion

The country experiences shared here provide evidence of the importance of lessons sharing to galvanize action and improve knowledge of ongoing work towards the 2030 agenda. Through such platforms, south-south collaboration across countries can be strengthened. While these interventions are not completely exhaustive, this paper has been designed to pull together high-level actions and lessons for policy consideration. Having these ideas presented through the experiences of countries in the African Region, action is within grasp to achieve UHC through PHC.
